# Assessing Machine Learning Models for Predicting Age with Intracranial Vessel Tortuosity and Thickness Information

**DOI:** 10.3390/brainsci13111512

**Published:** 2023-10-26

**Authors:** Hoon-Seok Yoon, Jeongmin Oh, Yoon-Chul Kim

**Affiliations:** Division of Digital Healthcare, College of Software and Digital Healthcare Convergence, Yonsei University, Wonju 26493, Republic of Korea; hoonseok123@yonsei.ac.kr (H.-S.Y.); ohjeongmin@yonsei.ac.kr (J.O.)

**Keywords:** machine learning, magnetic resonance angiography, intracranial artery, age prediction, medical image analysis, feature extraction

## Abstract

This study aimed to develop and validate machine learning (ML) models that predict age using intracranial vessels’ tortuosity and diameter features derived from magnetic resonance angiography (MRA) data. A total of 171 subjects’ three-dimensional (3D) time-of-flight MRA image data were considered for analysis. After annotations of two endpoints in each arterial segment, tortuosity features such as the sum of the angle metrics, triangular index, relative length, and product of the angle distance, as well as the vessels’ diameter features, were extracted and used to train and validate the ML models for age prediction. Features extracted from the right and left internal carotid arteries (ICA) and basilar arteries were considered as the inputs to train and validate six ML regression models with a four-fold cross validation. The random forest regression model resulted in the lowest root mean square error of 14.9 years and the highest average coefficient of determination of 0.186. The linear regression model showed the lowest average mean absolute percentage error (MAPE) and the highest average Pearson correlation coefficient (0.532). The mean diameter of the right ICA vessel segment was the most important feature contributing to prediction of age in two out of the four regression models considered. An ML of tortuosity descriptors and diameter features extracted from MRA data showed a modest correlation between real age and ML-predicted age. Further studies are warranted for the assessment of the model’s age predictions in patients with intracranial vessel diseases.

## 1. Introduction

Aging and its related diseases affect individuals during their life spans. Due to improvements in healthcare and medical technology, life expectancy and the proportion of elderly people have increased worldwide recently [1]. As a measure of brain-related aging, predictions of age from brain image data have been increasingly investigated by numerous research groups [2,3,4,5]. A great deal of studies has been conducted using structural magnetic resonance imaging (MRI) data for brain age prediction. Brain image data from healthy subjects have been used to develop a machine learning (ML) model for predicting chronological age [6,7]. T1-weighted structural brain MRI data have been used to train and validate a deep convolutional neural network (CNN) architecture [8]. Region-of-interest (ROI) volumes extracted from T1-weighted structural data have been used as features for ML regression models [9]. T1-weighted brain MRI and computed tomography (CT) data were used in a past study [10]. Multimodal MRI images, including T1-weighted imaging, T2* relaxometry, and diffusion tensor imaging (DTI) have been used to extract features and predict brain age using a multiple linear regression analysis [11]. T1-weighted, fluid-attenuated inversion recovery (FLAIR), and susceptibility-weighted imaging (SWI) images have been used to train a three-dimensional (3D) CNN architecture [12]. There have also been association studies between the morphometric features available from MRI image data and chronological age. Gray matter volume and fractional anisotropy tend to decrease with age [13]. High frequencies of white matter hyperintensities, microbleeds, or lacunar infarcts have been associated with aging [3].

MR angiography (MRA) or computed tomography angiography (CTA) provide insights different from those provided by commonly used structural T1-weighted brain image data. MRA or CTA image data facilitate the noninvasive assessment of vascular aging. Several studies have investigated the association between vascular tortuosity, kinking, and coiling and age [14,15,16]. A more detailed quantitative analysis of the cerebral vasculature can help assess chronological age [17]. The oldest age group tends to show a larger mean diameter of the major intracranial vessel than the youngest age group [17]. A lower number of unbranched small vessels has been observed in older age groups [17,18]. The length and meandering of bifurcating branches have been shown to increase with age [19], although the study did not involve an ML-based age prediction. Deep CNN models trained on healthy subjects’ MRA have been demonstrated to estimate the biological aging of intracranial vessels in patients suspected of having vessel-related diseases. Nam et al. proposed a deep CNN model for predicting chronological age using healthy subjects’ brain time-of-flight (TOF) MRA data [20]. Mouches et al. demonstrated high accuracy in brain age prediction by combining MRA data with T1-weighted data in deep CNN architectures [21]. They identified the basilar artery, the middle cerebral artery M2 segments, and the left posterior cerebral artery as the artery regions that mainly contribute to age prediction. It has been reported that arterial tortuosity is a risk factor for aneurysms or other vessel-aging-related diseases [14,22].

The extraction of morphometric features with a vessel analysis tool has been investigated to evaluate aging and disease conditions from the intracranial vasculatures [23]. With a similar purpose in mind, instead of using end-to-end deep CNN architectures, we proposed the use of tortuosity and diameter features extracted from a vessel segment in the region of the circle of Willis arteries from 3D TOF MRA image data, and we predicted subjects’ ages using an ML regression model. We also investigated what image features would contribute the most to the age predictions in some regression models.

## 2. Materials and Methods

### 2.1. Data and Preprocessing

Three-dimensional TOF MRA data and age information were available in the IXI datasets (https://brain-development.org/ixi-dataset (accessed on 25 October 2023)), which were obtained from normal and healthy subjects. After 87 subjects’ data were discarded, the data from 171 subjects were considered for our analysis. The data exclusion criteria were: (1) the image data were of poor quality, (2) the imaging field of view (FOV) was not sufficient along the superior-inferior direction to cover the basilar artery (BA) and internal carotid arteries (ICAs), or (3) the Dijkstra’s path-finding result was incorrect in either the left or right ICA. The data with the insufficient FOV were discarded in our analysis because the selection of the two endpoints needed to be consistent anatomically for the feature extraction. Also, the data with incorrect path-finding results were discarded because incorrect path-finding would result in incorrect feature extraction, which would negatively affect age prediction. The incorrect path-finding result was attributed to the fact that the Dijkstra algorithm finds the shortest path in a centerline, and a highly tortuous vessel such as the ICA may produce false positive centerlines which would be shorter than the correct path. The 171 subjects had a mean age of 49.3 years with a standard deviation of 16.5 years (age range = 20–83). A total of 85 subjects were male, and the remaining 86 subjects were female.

**Figure 1:**
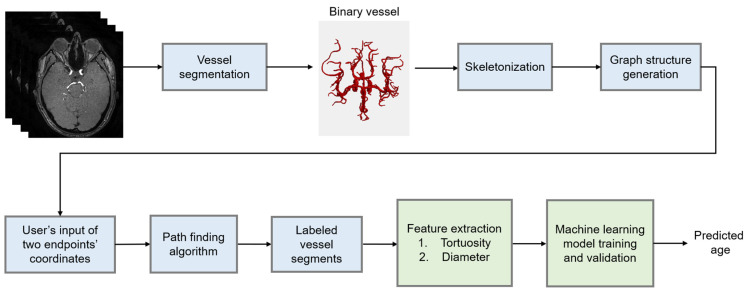
A flowchart of the overall process. The light green boxes are primarily related to the machine learning predictions of age.

**Figure 2:**
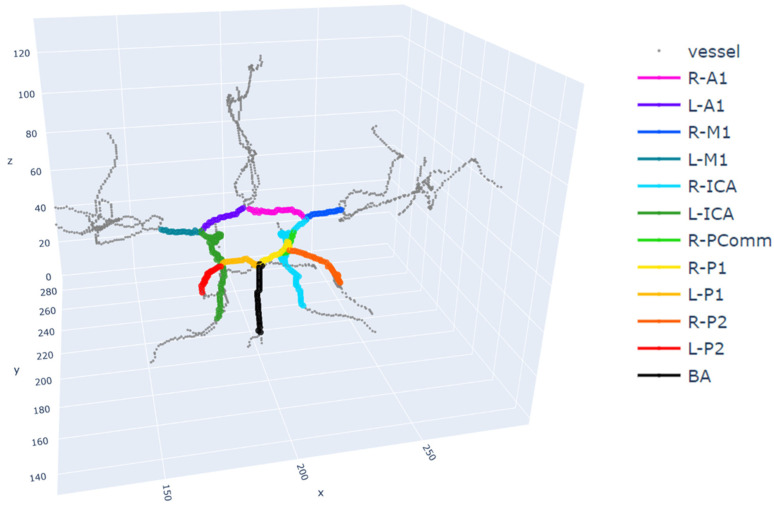
An example of the annotated vessel segments. A1, anterior cerebral artery (ACA) A1; M1, middle cerebral artery (MCA) M1; ICA, internal carotid artery; PComm, posterior communicating artery; P1, posterior cerebral artery (PCA) P1; P2, PCA P2; BA, basilar artery; R, right; L, left.

**Figure 3:**
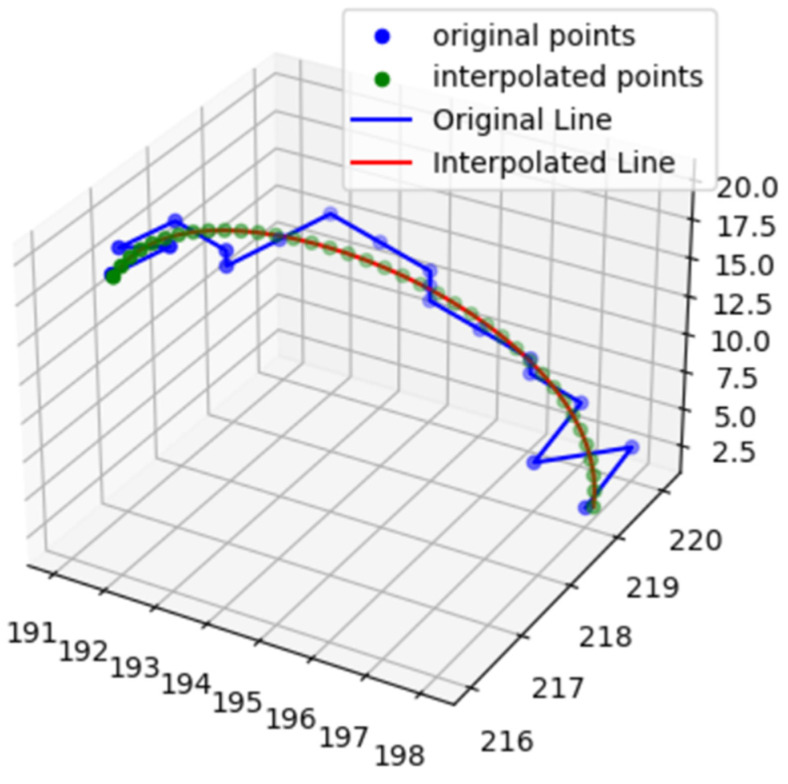
The spline interpolation for smoothing the centerline in a basilar artery. The skeleton is discretized in the 3D coordinate space, and hence, when displayed, it shows a jagged appearance, as indicated by the blue line. A spline interpolation results in the smooth curve which is indicated by the red line.

The overall process for the age predictions, including image preprocessing, is summarized in Figure 1. A three-dimensional seeded region-growing was performed to segment the vessels. The morphology.skeletonize function of the Scikit-Image library [24] was used to obtain the 3D centerlines of the vessels. The Plotly (Plotly Technologies Inc., Montreal, QC, Canada) Python library (https://plotly.com/python (accessed on 25 October 2023)) was used to manually annotate the landmarks in the circle of Willis arteries [25]. The Dijkstra algorithm was used to find a path between two manually annotated endpoints. The vessel segments were labeled with colors, as shown in Figure 2, and they included the anterior communicating artery (ACOMM), left and right anterior cerebral arteries (L-A1 and R-A1, respectively), left and right middle cerebral arteries (L-M1 and R-M1, respectively), left and right internal carotid arteries (L-ICA and R-ICA, respectively), left and right posterior communicating arteries (L-PCOMM and R-PCOMM, respectively), left and right posterior cerebral arteries (L-P1, R-P1, L-P2, and R-P2, respectively), and basilar arteries (BA). In Figure 2, ACOMM and L-PCOMM are not shown since the centerlines of these vessel segments were not shown after the vessel segmentation followed by the skeletonization. The resulting segments of the centerlines were colored differently, and they were saved in .html files, which were opened to verify the accuracy of the manual annotations. The points along the centerline underwent smoothing via spline interpolation, as indicated by the red line in Figure 3.

### 2.2. Feature Extraction

The extracted features for each segment consisted of the curve length ($l_{c}$), Euclidean length (l), relative length (RL), sum of angle metrics (SOAM), product of angle distance (PAD), and triangular index (TI). Table 1 shows the descriptions and mathematical formulations for the tortuosity-related features along with image illustrations. In addition, diameters were calculated along the centerline points using the ndimage.distance_transform_edt function in the SciPy library [26] on a 3D binary vessel mask. The mean, minimum, maximum, standard deviation, 25th percentile, 50th percentile, and 75th percentile values of the diameters were extracted for each segment. These features for the arterial segments in all subjects considered were saved as .xlsx files. Some subjects’ data had missing values in certain arterial segments. In our study, the ACOMM, L-PCOMM, and R-PCOMM segments had more missing data than the remaining vessel segments.

To develop the ML models for age estimation, we took features from a subset of the artery segments. The subset consisted of the left and right internal carotid artery (ICA) tortuosity descriptors and basilar artery (BA) tortuosity descriptors, as well as the left and right ICA and BA diameter features. We determined two endpoints in a left or right ICA vessel segment by annotating one endpoint at the bifurcation of the ACA, ICA, and MCA and annotating the other endpoint at the highest curvature of the C3 segment. We also determined two endpoints in a BA vessel segment by annotating one endpoint at the bifurcation of the BA and PCAs and annotating the other endpoint at the bifurcation of the BA and vertebral arteries (VAs). The BA and ICAs were chosen because there were no missing data in their feature values across all subjects, and they had relatively greater vessel lengths and thicknesses than the other vessel segments, facilitating a more reliable quantitative image analysis than the small and thin vessel segments whose vessel diameters could be close to the width of one or two voxels. Hence, the total number of features per subject was 3 × 13 = 39, which is equivalent to three vessel segments multiplied by 13 features (i.e., six features related to tortuosity and seven features related to vessel diameter statistics).

### 2.3. Model Development

The ML model development was performed using the Scikit-learn library [27]. We compared the following six ML models for age prediction: (1) random forest regression, (2) linear regression, (3) AdaBoost regression, (4) gradient boosting regression, (5) Bayesian ridge regression, and (6) XGBoost regression.

#### 2.3.1. Random Forest

Random forest is an ensemble-based ML method that uses the predictions made by multiple decision trees. Each decision tree is trained on data samples randomly selected with replacements [28]. Moreover, a subset of features is randomly chosen for a decision tree during training. In regression, a test sample goes through all the trained decision tree models individually, and then the final output is the average of the predictions made by the decision tree models. Random forest is popular due to its simplicity for training.

#### 2.3.2. Linear Regression

In a linear regression model, the output is given as a linear combination of the input features with a bias term added. Linear regression is simple and interpretable because it can describe how the inputs affect the output. Training is performed based on parameter estimation on a linear model with a certain optimization method. The least squares method is the most popular method for the parameter estimation [29]. The linear model parameters are used to predict an outcome with test data. With regards to prediction performance, linear regression sometimes can be superior to other sophisticated nonlinear ML models in cases with small numbers of training samples or low signal-to-noise ratio samples.

#### 2.3.3. Bayesian Ridge Regression

A Bayesian ridge regression model is a model that utilizes a posterior probability distribution in its weight parameters. Bayesian ridge regression adds the l2-norm regularization terms to the objective function of the linear regression model. The model parameters in the linear model are assumed to follow a Gaussian distribution [30]. Bayesian ridge regression with l2-norm regularization not only prevents overfitting but also is robust against outliers.

#### 2.3.4. AdaBoost

AdaBoost is an acronym for adaptive boosting algorithm [31]. The boosting first generates many weaker ML classifiers during training, and then it creates a final model by constructing a fine-tuned and stronger classifier with all the weaker models. It trains a base classifier from an initial training dataset and then modifies the weights of the initial training data samples based on the classifier’s performance. The weights of the misclassified samples become larger, and a new base classifier will be trained with the samples whose weights are changed. Among ML models, AdaBoost is less prone to overfitting and can be used to improve the accuracy of weaker classifiers.

#### 2.3.5. Gradient Boosting

Gradient boosting is an ML approach that first generates a decision tree to approximate a non-linear relationship between the input features and the output, and then it uses boosting as described in the AdaBoost method. It works by iteratively fitting a new model to the residual errors of a previous model. Unlike AdaBoost, gradient boosting updates weights using gradient descent. The method can reduce bias, but overfitting may occur. It theoretically performs better than AdaBoost, but it takes computationally longer and has a memory disadvantage.

#### 2.3.6. XGBoost

XGBoost is an acronym for extreme gradient boosting [32]. It is based on gradient boosting, and importantly, it is designed to support parallel computing for time-efficient predictions from large scale datasets. It learns a tree model during each training iteration to minimize residual errors. XGBoost includes regularization to prevent overfitting and is widely adopted in medical data analysis due to its improved performance in terms of speed, scalability, and accuracy.

#### 2.3.7. Training and Validation

A four-fold cross-validation was performed for our evaluation. The 171 subjects were randomly assigned to one of the four groups for the cross-validation. For each fold, hyperparameters were tuned on the training data using a randomized search [33] after the determination of a candidate hyperparameter set in the scikit-learn library. Table 2 shows the hyperparameter values used to train each of the following five regression models: random forest regression, AdaBoost regression, gradient boosting regression, Bayesian ridge regression, and XGBoost. An evaluation based on the validation data was performed using the metrics described in the next section.

### 2.4. Evaluation

The performance of the age prediction was quantified using the below metrics. We assumed that $x_{i}$ was the real chronological age of the *i*th subject and $y_{i}$ was the predicted age of the *i*th subject. We defined the Pearson correlation coefficient as follows:

(1) $$r=\frac{\sum_{i=1}^{N}\left(x_{i}-\bar{x})(y_{i}-\bar{y}\right)}{\sqrt{\sum_{i=1}^{N}{\left(x_{i}-\bar{x}\right)}^{2}\sum_{i=1}^{N}{\left(y_{i}-\bar{y}\right)}^{2}}},$$

where $\bar{x}$ is a mean of the $x_{i}$ values and $\bar{y}$ is a mean of the $y_{i}$ values. We defined the root mean square error (RMSE) as follows:

(2) $$RMSE=\sqrt{\frac{\sum_{i=1}^{N}{\left(x_{i}-y_{i}\right)}^{2}}{N}}.$$

We defined the mean absolute percentage error (MAPE) as follows:

(3) $$MAPE=\frac{1}{N}\sum_{i=1}^{N}\left|\frac{x_{i}-y_{i}}{x_{i}}\right|.$$

The coefficient of determination or $R^{2}$ was defined as follows:

(4) $$R^{2}=1-\frac{\sum_{i=1}^{N}{\left(x_{i}-y_{i}\right)}^{2}}{\sum_{i=1}^{N}{\left(x_{i}-\bar{x}\right)}^{2}}$$

Feature importance was calculated for the tree-based models, including the random forest regression, Adaboost regression, gradient boosting regression, and XGBoost regression models with the Scikit-learn library. The calculation was based on the contribution of each feature to the model’s prediction performance. The features that led to significant reductions in the Gini impurity or mean square error were considered more important.

Qualitative evaluation was performed by visualizing the scatter plot, which showed correlations between the chronological ages and the ML-predicted ages. Its implementation was made using the Matplotlib Python library [34].

## 3. Results

After applying the data exclusion criteria provided in the Materials and Methods section, 87 subjects’ data were discarded. The remaining 171 subjects were considered for analysis. Table 3 shows the prediction performances of the six ML regression models. The random forest regression model exhibited the lowest average RMSE and the highest average R^2^. The linear regression model exhibited the lowest average MAPE and the highest average Pearson correlation coefficient. The Bayesian ridge regression model showed the poorest performance in all the evaluation metrics considered in this study. The AdaBoost and XGBoost regression models showed similar overall performances. The AdaBoost regression model ranked second for its RMSE, third for its R^2^, third for its MAPE, and fourth for its Pearson correlation coefficient. The XGBoost regression model ranked fourth for its RMSE, fourth for its R^2^, second for its MAPE, and third for its Pearson correlation coefficient.

**Figure 4:**
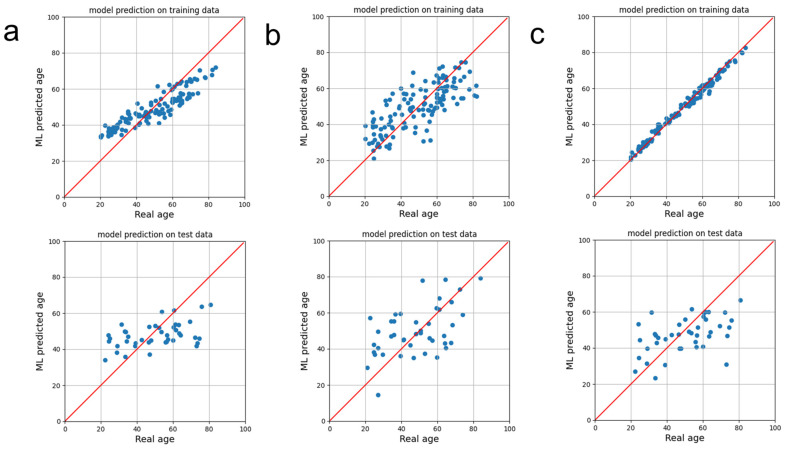
Scatter plots of the age predictions of the (**a**) random forest regression, (**b**) linear regression, and (**c**) XGBoost regression models. The top plots are models’ predictions on the training data, and the bottom plots are the models’ predictions on the test data. The blue dots are samples, and the red line is the y = x line.

Figure 4 shows the scatter plots for the real ages vs. the ML-predicted ages for the exemplary training and the validation data for three regression models, which were (a) the random forest regression model, (b) the linear regression model, and (c) the XGBoost regression model. The random forest model showed overestimation in the young age range while showing underestimation in the old age range, as shown in the training data plot in Figure 4a. The linear regression model showed a more highly scattered plot than the other two models when the left figure in Figure 4b is compared with the left figures in Figure 4a,c. The XGBoost regression model showed almost perfect alignment along the y = x line, as shown in the top figure of Figure 4c, but the validation result showed a more scattered result than the training result, as shown in Figure 4c, implying overfitting in the model training procedure.

**Figure 5:**
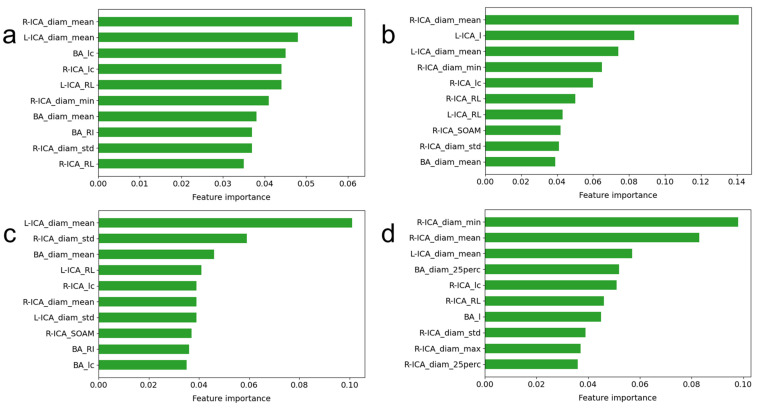
Lists of the ten most important features for the (**a**) random forest regression, (**b**) AdaBoost regression, (**c**) gradient boosting regression, and (**d**) XGBoost regression models.

Figure 5 shows the ten most important features estimated from the four tree-based models of the random forest regression, AdaBoost regression, gradient boosting regression, and XGBoost regression models. The R-ICA_diam_mean ranked first in the random forest regression and AdaBoost regression models. It ranked second in the XGBoost regression model and sixth in the gradient boosting model. The L-ICA_diam_mean was also one of the important contributing factors in all four models. It ranked second in the random forest regression model, third in the AdaBoost regression model, first in the gradient boosting regression model, and third in the XGBoost regression model. The R-ICA_diam_mean, L-ICA_diam_mean, R-ICA_lc, and R-ICA_diam_std features were in the top-ten list in all four models. The diameter-related features appeared more in the list than the tortuosity-related features for the XGBoost regression model, as shown in Figure 5d.

## 4. Discussion

We provided the first demonstration of ML-based age prediction and a feature importance assessment based on angiography image data with tortuosity and thickness features extracted from individual vessel segments in the ICA and BA. A variety of ML regression models were trained and validated, and they demonstrated different age prediction scores in terms of RMSE, R^2^, MAPE, and Pearson correlation coefficient values. Our findings indicated that the linear regression model showed a prediction performance similar to or higher than the other complex ML models in certain evaluation metrics, although the other complex ML models are known to be well-suited for data showing a nonlinear relationship between features and outcomes. In addition, the ML prediction performance was not as high as expected for the tortuosity and thickness features. This implied that vessel geometry features may not be the determining factors for predicting chronological ages in healthy volunteers. It is noted from the literature that confounding factors such as aerobic activity level can make it difficult to predict chronological age by relying on a vessel’s morphometric features alone for ML prediction [35].

We considered the ICAs and BA segments for age prediction. The reason why we chose these segments was that these vessels are usually longer and thicker than other vessel segments such that it could be easier to perform a reliable quantification of vessel tortuosity and diameter measurements. There may be room for improvement in age prediction by considering other vessel segments with longer vessel lengths than those used in our study. For example, Wright et al. analyzed PCAs, ACAs, and MCAs, including their distal branches, in order to associate their morphometric measurements with age. They demonstrated a Pearson correlation coefficient of 0.30 between age and bifurcating branch length, a Pearson correlation coefficient of 0.18 between age and fractal dimension, and a Pearson correlation coefficient of 0.44 between age and tortuosity [19]. Bullitt et al. indicated that the number of unbranched vessels was lower for an older age group. They interpreted their results by stating that as a result of the thickening of vessel wall due to aging, the lumen of the small vessel became too thin to be imaged for the old age group [17]. Chen et al. [23] suggested a detailed analysis by tracing more distal arteries and labeling arteries in more precise categories than the method used in the study of Wright et al. [19].

A 3D CNN model trained using MRA image data was investigated for the purpose of predicting age [20]. Unlike our approach, their method was an end-to-end approach that took a 3D TOF MRA image as an input and the predicted age as the output. In contrast to the deep CNN model development and validation, our approach was more direct in explaining what features contributed the most to age prediction. The deep CNN model automatically finds features from the data itself, but one of the major concerns is its black-box nature, which lacks in the model’s interpretability of the prediction results. Our study evaluated the rankings of feature importance in four tree-based ML models and indicated that the mean vessel diameter information in the right ICA segment was the highest-contributing feature for age prediction.

There was a similar study that investigated the performance of age prediction using ML regression models. Simfukwe and Youn applied ML regression models to features of brain region volumes in order to predict brain age [36]. In their study, the Bayesian ridge regression model showed the highest R^2^ value of 0.3 compared to other regression models, including the gradient boosting regression, support vector regression, and linear regression models. In our study, the extracted tortuosity and diameter features could be used for other prediction purposes. One potential area of application is risk prediction for intracranial aneurysm, as demonstrated by Nouri et al. [37].

The proposed method involved image segmentation, feature extraction, and ML-based prediction. Automatic segmentation based on deep learning may help improve the object or lesion identification performance [38,39]. Radiomic feature extraction could increase the dimensions of the features for precision medicine [40]. We focused on age prediction, but this framework could be applied to other purposes such as diagnosis [41], clinical decision-making [42], and outcome prediction [43] in acute stroke neuroimaging and tumor imaging.

SHapley Additive exPlanations (SHAP) [44] was not applied to our study, though it has the potential to interpret a machine learning model by finding a distribution of the contribution of each feature value in the model’s outcome. The feature with the widest distribution has the highest impact, and thus, it is regarded as the most important feature. SHAP has numerous medical applications in interpreting a predictive machine learning model. For example, its interpretability has been demonstrated in a prognostic risk stratification model for acute ischemic stroke [45], a feature selection task for the diagnosis of Parkinson’s disease [46], and a classification task for the diagnosis of glaucoma [47].

Our study had several limitations. First, the data used in our study did not have clinical information such as history of stroke, smoking, hypertension, dyslipidemia, etc. The predictions were made solely using angiographic image features. Second, the sample size was small, and hence, the data did not encompass substantial biological and anatomical variations among the subjects. Third, the data used in our study had static measurements and did not reveal temporal changes that occurred over an individual’s lifetime. Fourth, our image analysis approaches were sophisticated such that it was difficult to obtain large-scale data from multiple institutions. They involved multiple steps for image preprocessing such that any error in any processing step would result in invalid data for the training and validation of a machine learning prediction model.

## 5. Conclusions

This study showed the feasibility of age prediction using tortuosity and vessel diameter features extracted from individual vessel segments in circle of Willis arteries in magnetic resonance angiography data. Six machine learning regression models were developed and validated for age prediction. The linear regression model showed the highest correlation between real age and predicted age. A drawback of the proposed approach was the need for manual annotations of individual vessel segments as these are time consuming. The proposed method based on an ML regression of arterial segments’ vessel features had the advantage of explicitly determining the importance of features, while deep CNN prediction on an MRA image lacks interpretability. The proposed semi-automatic technique for quantifying segmental blood vessels’ morphometric features on TOF-MRA images is viable not only for age prediction, but also for other research areas such as the evaluation of treatment effects in atherosclerotic vessels and the assessment of risk of aneurysm in the bifurcations of vessels.

## Figures and Tables

**Table 1:** List of tortuosity features per vessel segment.

Feature Name	Description	Illustration
Curve length ($l_{c}$)	The length along the curve	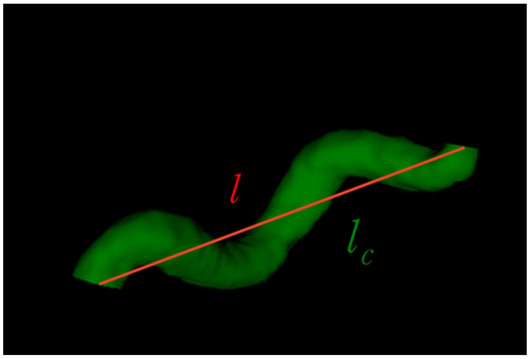
Euclidean length (l)	The distance between starting and ending points of the curve	
Relative length (RL)	$RL=\frac{l}{l_{c}}$	
Sum of angle metrics (SOAM)	$\frac{\sum_{i=1}^{n}\left(180{}^{\circ}-\varphi_{i}\right)}{l_{c}}$ $\varphi_{i}$: the angle between two adjacent lines connecting the center and two endpoints for the *i*th center	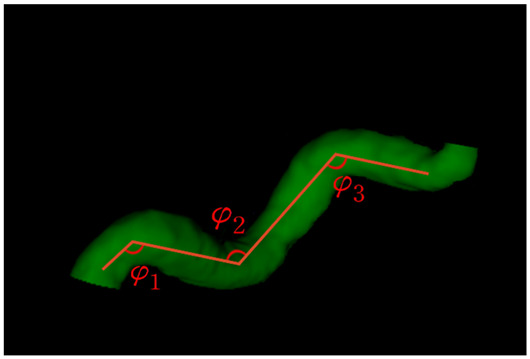
Product of angle distance (PAD)	$PAD=\frac{SOAM}{RL}$	
Triangular index (TI)	$TI=\frac{\sum_{i=1}^{n}\frac{a_{i}+b_{i}}{c_{i}}}{n}$	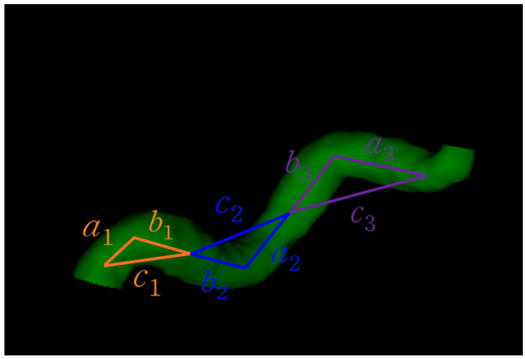

**Table 2:** Hyperparameter settings of the regression models.

Regression Model	Hyperparameter
Random forest regression	n_estimators = 74, max_depth = 14, min_samples_leaf = 2, min_samples_split = 6, max_features = ‘sqrt’, criterion = ‘absolute_error’
AdaBoost regression	learning_rate = 0.07, loss = ‘exponential’, and n_estimators = 163
Gradient boosting regression	criterion = ‘friedman_mse’, learning_rate = 0.11, loss = ‘absolute_error’, max_depth = 15, n_estimator = 198, and tol = 0.003
Bayesian ridge regression	alpha_1 = 0.0004, alpha_2 = 1 × 10^−6^, alpha_init = 10, lambda_1 = 1 × 10^−5^, and Tol = 0.01
XGBoost regression	colsample_bytree = 0.95, gamma = 0.22, learning_rate = 0.30, max_depth = 4, n_estimators = 138, and subsample = 0.84

**Table 3:** Evaluation of the models’ predictions using the four-fold cross-validation. The bold text indicates the best performances among the models.

Regression Model	Root Mean Squared Error (RMSE)	R^2^	Mean Absolute Percentage Error (MAPE)	Pearson Correlation Coefficient
Random forest regression	**14.867 ± 0.515**	**0.186 ± 0.031**	0.319 ± 0.022	0.459 ± 0.058
Linear regression	15.000 ± 1.032	0.162 ± 0.140	**0.290 ± 0.015**	**0.532 ± 0.067**
AdaBoost regression	14.965 ± 0.496	0.175 ± 0.029	0.315 ± 0.023	0.438 ± 0.044
Gradient boosting regression	15.554 ± 1.478	0.106 ± 0.136	0.320 ± 0.034	0.357 ± 0.163
Bayesian ridge regression	16.009 ± 0.419	0.055 ± 0.041	0.347 ± 0.018	0.258 ± 0.089
XGBoost regression	15.207 ± 0.905	0.148 ± 0.062	0.312 ± 0.031	0.456 ± 0.072

## Data Availability

The data presented in this study are available on request from the corresponding author.
